# Facile fabrication of sulfonated porous yeast carbon microspheres through a hydrothermal method and their application for the removal of cationic dye

**DOI:** 10.1038/s41598-024-62283-w

**Published:** 2024-05-17

**Authors:** Yang Chenxi, Zhang Haiou, Wang Jian, Wang Yingguo

**Affiliations:** 1 Institute of Land Engineering and Technology, Shaanxi Provincial Land Engineering Construction Group Co., Ltd, Xi’an, 710075 China; 2 ShaanXi Provincial Land Engineering Construction Group Co., Ltd., Xi’an, 710075 China; 3 Key Laboratory of Degraded and Unused Land Consolidation Engineering, The Ministry of Natural Resources. Ltd., Xi’an, 710075 China; 4 Shaanxi Provincial Land Consolidation Engineering Technology Research Center, Xi’an, 710075 China

**Keywords:** Yeast, Hydrothermal, Adsorbent, Cationic dye, Environmental chemistry, Chemistry

## Abstract

Water pollution containing dyes become increasingly serious environmental problem with the acceleration of urbanization and industrialization process. Renewable adsorbents for cationic dye wastewater treatment are becoming an obstacle because of the difficulty of desorbing the dye from the adsorbent surface after adsorption. To overcome this dilemma, herein, we report a hydrothermal method to fabricate sulfonic acid modified yeast carbon microspheres (SA/YCM). Different characterization techniques like scanning electron microscopy, FTIR spectroscopy, and X-ray diffraction have been used to test the SA/YCM. Decorated with sulfonic acid group, the modified yeast carbon microspheres possess excellent ability of adsorbing positively charged materials. The removal rate of Methyl blue (MB) by renewable adsorbent SA/YCM can reach 85.3% when the concentration is 500 mg/L. The SA/YCM regenerated by HCl showed excellent regeneration adsorption capacity (78.1%) after five cycles of adsorption–desorption regeneration experiment. Adsorption isotherm and kinetic behaviors of SA/YCM for methylene blue dyes removal were studied and fitted to different existing models. Owing to the numerous sulfonic acid groups on the surface, the SA/YCM showed prominent reusability after regeneration under acidic conditions, which could withstand repeated adsorption–desorption cycles as well as multiple practical applications.

## Introduction

Due to the high toxicity and potential accumulation in the environment, water pollution containing dyes become increasingly serious environmental problem with the acceleration of urbanization and industrialization process^1–4^. In recently, there are over 100,000 commercial dyes globally, and approximately 280,000 tons of these dyes are released into water systems annually. These organic molecules possess potential toxicity, carcinogenicity, teratogenicity, and non-biodegradability properties, rendering them hazardous environmental pollutants^5^. Most of these dyes classified based on their charged properties into three different classes as cationic dyes, anionic dyes, and neutral dyes^6–9^. Most of these dyes contain aromatic rings, which cause them carcinogenic and mutagenic^10,11^. As a result of the intensive use of these dyes in industry, they are an integral part of industrial wastewater. Consequently, the removal of dyes from wastewater is currently of great interest. For solving these dye wastewater problems, a great deal of physical, chemical, and biological methods of treatment of dye wastewater have been attempted. These include adsorption methods^12,13^, membrane separation technology^14,15^, oxidation processes^16,17^, photocatalytic degradation^18,19^ et al. Among the advanced chemical or physical treatments, adsorption is considered to be a promising technology for solving dye wastewater. Owing to the merits of easy availability, simplicity of design, easy operation and the ability to process dyes in more concentrated forms, some traditional materials with adsorption properties including activated carbon^20,21^, biochar^22,23^, and graphene^24,25^ have been currently utilized for the dye wastewater treatment. The prominent dye removal capacity of these absorbents was due to the merits of high porosity and large specific surface area^26–28^.

A large amount of pollutants (dyes, petroleum, heavy metals, pathogen et al.) are adsorbed simultaneously on the surface of the absorbent, which reduces the single pollutant adsorption capacity of the absorbent^29,30^. Due to the complex composition of pollutants in water, adsorbents with the ability to adsorb target pollutants are suitable for removal of a large amount of certain pollutants. Sticking to these principles, carbon microsphere are effective absorbents for overcoming this dilemma ^31^. Carbon microspheres are highly popular due to their unique properties, such as a high aspect ratio, and high thermal, mechanical and electrical properties^32–34^. The high aspect ratio of carbon microsphere, makes them a viable option for water treatment ^35^. The high porosity and large surface area of carbon microspheres provide ample adsorption active sites for harmful cations, anions and other inorganic and organic contaminants found in wastewater ^36^. Hao et al. ^37^. prepared carbon microspheres (CSn) with various oxygen-containing functional groups (−OH, − C═O, −COOR) from factory poplar waste. The impact of various adsorption parameters, including adsorption duration, temperature, solution pH, and ion concentration, was analyzed. The results suggest that the quantity of oxygen-containing functional groups in CSn diminished with the rise in temperature, consequently reducing the adsorption capacity for methylene blue. Deng et al.^38^. proposed the fabrication of nitrogen-containing chitosan-based porous carbon microspheres (CPCM) using HCl and KOH. The spherical morphology and honeycomb-like porous structure of CPCM were precisely controlled. A significant number of micro/mesopores resulted in an exceptionally high specific surface area for CPCM, reaching up to 2463.9 m^2^ g^−1^. Additionally, CPCM demonstrated an outstanding maximum adsorption capacity of 1599.03 mg g^−1^ for methylene blue (MB). However, it has been proved that these absorbents still have limitation for dyes with different electric properties. For instance, carbon microspheres can only passively absorb dyes in water, and cannot rapidly reduce the concentration of dyes in water. Further, the desorption of carbon microspheres after dye adsorption is slow, which seriously affects the sustainable use of adsorbent. Accordingly, it is significant to fabricate the absorbent with good porosity, active dye adsorption ability and low-cost.

Herein, yeast, an economical, accessible, and safe microorganism with a lot of polysaccharides on cell wall and containing plentiful reactive groups (hydroxyl, carboxylic acid, amine, phosphate, and amide) on the surface^39,40^, was elaborately selected as a substrate to synthesize a new excellent cationic dye absorbent through attaching the sulfonic acid group onto the yeast surface. Concurrently, the hydrothermal process turns yeast into carbon microspheres with greater porosity. The prominent cationic dye adsorption performance of these sulfonic acid modified yeast carbon microspheres lies in the porosity and more negative charges on the surface. More importantly, these advanced yeast carbon microspheres showed prominent reusability, and could withstand repeated adsorption–desorption cycles due to the good mechanical properties of the carbon microsphere and the strong adhesion of the sulfonic acid groups. This example of sulfonic acid modified yeast carbon microspheres can be exploited as a smart sorbent to realize rapid treatment of cationic dye wastewater by a simple adsorption method.

## Experimental section

### Material

Dry yeast was obtained from Angel Yeast Co., Ltd (Yichang City, Hubei Province). Ethanol was supplied by Tianjin Chemical Reagent Factory (Tianjin, China). 2-hydroxyethanesulphonic acid was purchased from Macklin (Shanghai, China). The above-mentioned materials are used directly without purification.

### Preparation of sulfonated porous yeast carbon microspheres

The typical hydrothermal preparation process was as follows: 2.0 g of dry yeast was cleaned in 200 mL of 99.5% ethanol and deionized water for 1.0 h to remove ions and organics from the yeast. Afterwards, 2.0 g of 2-hydroxyethanesulphonic acid was added to 60 mL of water and magnetically stirred for 1.0 h. Further, the as–obtained solution and yeast were added to a 100 mL polytetrafluoroethylene autoclave. Sulfonic acid modification was maintained at 200 °C for 4.0 h. The obtained materials were soaked in methanol (100 mL) and deionized water (100 mL), and clean the as-obtained material with magnetic stirring for 2 min (100 r/min) to eliminate the remaining chemicals. The washed samples were dried under environmental conditions to produce the prepared material. Ultimately, the sulfonic acid modified yeast carbon microspheres were successfully prepared.

### Characterization

The as-obtained sample was ground with a mortar and taken 2 mg. Subsequently, The Fourier transform infrared (FTIR) spectra of the as-obtained samples were analyzed in KBr using a PerkinElmer FTIR spectrometer in the range of 4000–500 cm^–1^. The microstructures of as-obtained materials were investigated, and 0.2 cm^2^ was cut. The scanning electron microscope (SEM) (Hitachi S-4800) was used for observation after gold plating for 2 min. Before XRD characterization, weigh approximately 0.5 g of the sample and place it in an agate mortar for thorough grinding until no noticeable graininess remains when pinching the sample with fingers. Place the ground powder sample into the groove of the glass sample holder, and compact it evenly with a glass slide to ensure that the sample surface is uniform, flat, and on the same horizontal plane as the edge of the sample holder. The crystalline structure of as-obtained samples were determined by X-ray diffraction (XRD) using a Rigaku (RINT 2000) diffractometer. The surface area, as determined by the Brunauer–Emmett–Teller (BET), and the pore size distribution, as assessed using the Barrett–Joyner–Halenda (BJH), were derived from the adsorption/desorption data of the samples. The UV–vis spectra were obtained by the PerkinElmer UV–Vis (Lambda 750) spectrophotometer. First, prepare the liquid sample for testing and position it within the spectrometer's sample chamber. Adjust the spectrometer's wavelength to 630 nm. Then, determine the dye concentration in the liquid by using the sample's absorbance value in conjunction with the standard curve.

### Measurements of equilibrium adsorption of methylene blue

To investigate the ability of SA/YCM to remove methylene blue (MB) from wastewater, the adsorption experiment of the as–prepared SA/YCM was determined by UV–vis spectrophotometer. The adsorption capacity of as–prepared material was determined according to the procedure as aforementioned above and was calculated according to the following equation:

1 $${q}_{e}=\frac{({{C}}_{o}-{C}_{e}){V}}{{m}}$$

2 $${q}_{t}=\frac{({C}_{o}-{C}_{t}){V}}{{m}}$$

3 $$q\left(\%\right)=\frac{\left({C}_{o}-{C}_{e}\right)}{{C}_{o}}\times 100\%$$

where *q*_e_ and *q*_t_ represent the equilibrium adsorption amount and instantaneous adsorption amount, respectively. *C*_0_, *C*_e_ and *C*_t_ are the initial concentration, adsorption equilibrium concentration and instantaneous concentration, respectively. *V* is the volume of solution, m is the weight of adsorbent material.

To further investigate the effect of temperature on MB removal, thermodynamic models were calculated according to the following equation:

4 $$K_{{\text{c}}} = q_{{\text{e}}} /C_{{\text{e}}}$$

5 $$\Delta G = \, - RT{\text{ln}}\,K_{{\text{c}}}$$

6 $${\text{ln}}\,K_{c} = \Delta S/R - \Delta H/RT$$

where *R* is the molar gas constant; *K*_c_ is the adsorption equilibrium constant; *T* is the absolute temperature (K); *C*_e_ represent the reaction equilibrium concentration (mg/L); and Δ*G*, Δ*S*, and Δ*H* is the changes in Gibbs free energy (KJ/mol), entropy [J/(molK)], and enthalpy (kJ/mol)^36^.

## Result and discussion

### Formation of SA/YCM

**Figure 1:**
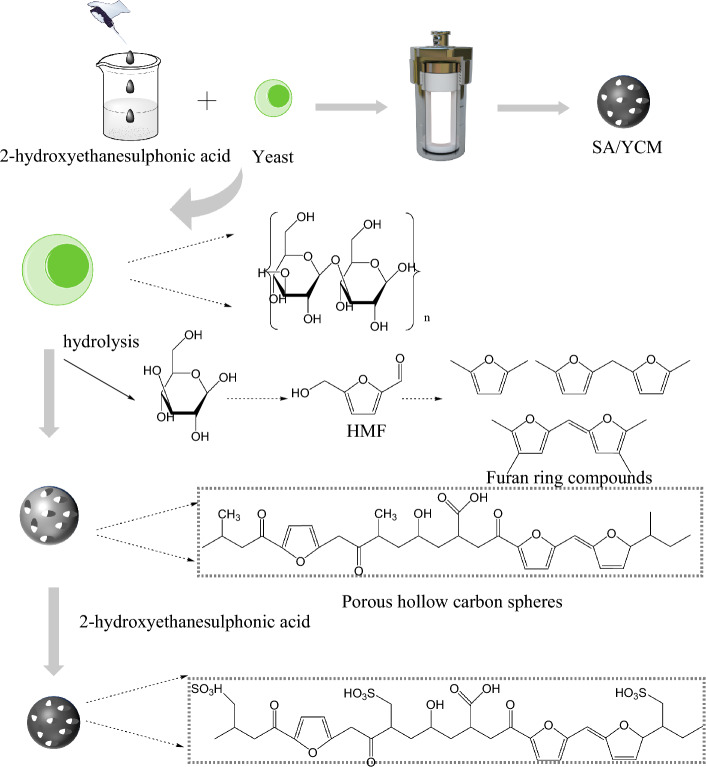
Schematic illustration of the preparation of SA/YCM via hydrothermal process and its chemical change mechanism.

As shown in Fig. 1, sulfonated porous yeast carbon microspheres were synthesized using a simple hydrothermal method. The hydrothermal synthesis of carbon microspheres involves the generation of carbon materials through the reaction of carbon sources with other raw materials under high-temperature, high-pressure hydrothermal conditions. This process is characterized by its simplicity and ease of operation, as well as a rapid and controllable reaction rate. This as-obtained carbon sources should be very worthy for the cationic dye adsorption and water retention. First, yeast possesses unique properties, i, e., it is commercially available, cost-effective, and environmentally friendly (e.g., nontoxic)^41,42^. What’s more, the polysaccharide network of the yeast cell wall consists of an amorphous matrix and fiber network^21^. The amorphous matrix is sensitive to hydrolysis and the fiber network is resistant to decomposition. Accordingly, yeast was selected as the substrates because it is commercially available and easy to carbonize, which inevitably endows it with excellent advantages for subsequent modification. Second, the fibril network of yeast tends to dehydrate within the molecule to form the scaffold of carbon microspheres under hydrothermal conditions, while the amorphous matrix undergoes severe decomposition through hydrolysis and is converted into monosaccharides and oligosaccharides. Further, the hydrolysis of monosaccharides and oligosaccharides yields 5-hydroxymethylfurfural (HFM) intermediates, which are then combined to the porous carbon surface through a series of reactions (decomposition, polymerization and condensation), to form furan ring compounds^43,44^. In this process, the competition between carbonization and hydrolysis determines the final form of porous carbon. Small hydrolysis channels appear in some shells due to the uneven mass transfer resistance in the hydrolysis process. During further hydrothermal treatment, dehydration occurs, and a large amount of gas in the cell needs to penetrate the microsphere shell to form pores, leading to the production of porous carbon. Third, the sulfonic acid groups, were firmly attached to the surface of the yeast carbon by hydrothermal process. More specifically, hydroxyethyl sulfonic acid with hydroxyl active functional group can be dehydrated and polymerized with the hydroxyl group on the surface of HFM to form a micro carbon containing sphere with sulfonic acid group. Subsequently, the loss of water from these components led to the further aggregation of microspheres into larger spheres, which eventually produced sulfonated porous yeast carbon microspheres. As expected, the porous yeast carbon microspheres with sulfonic acid groups had good cation adsorption characteristics and water absorption capacity.

### Surface morphology and chemical composition

**Figure 2:**
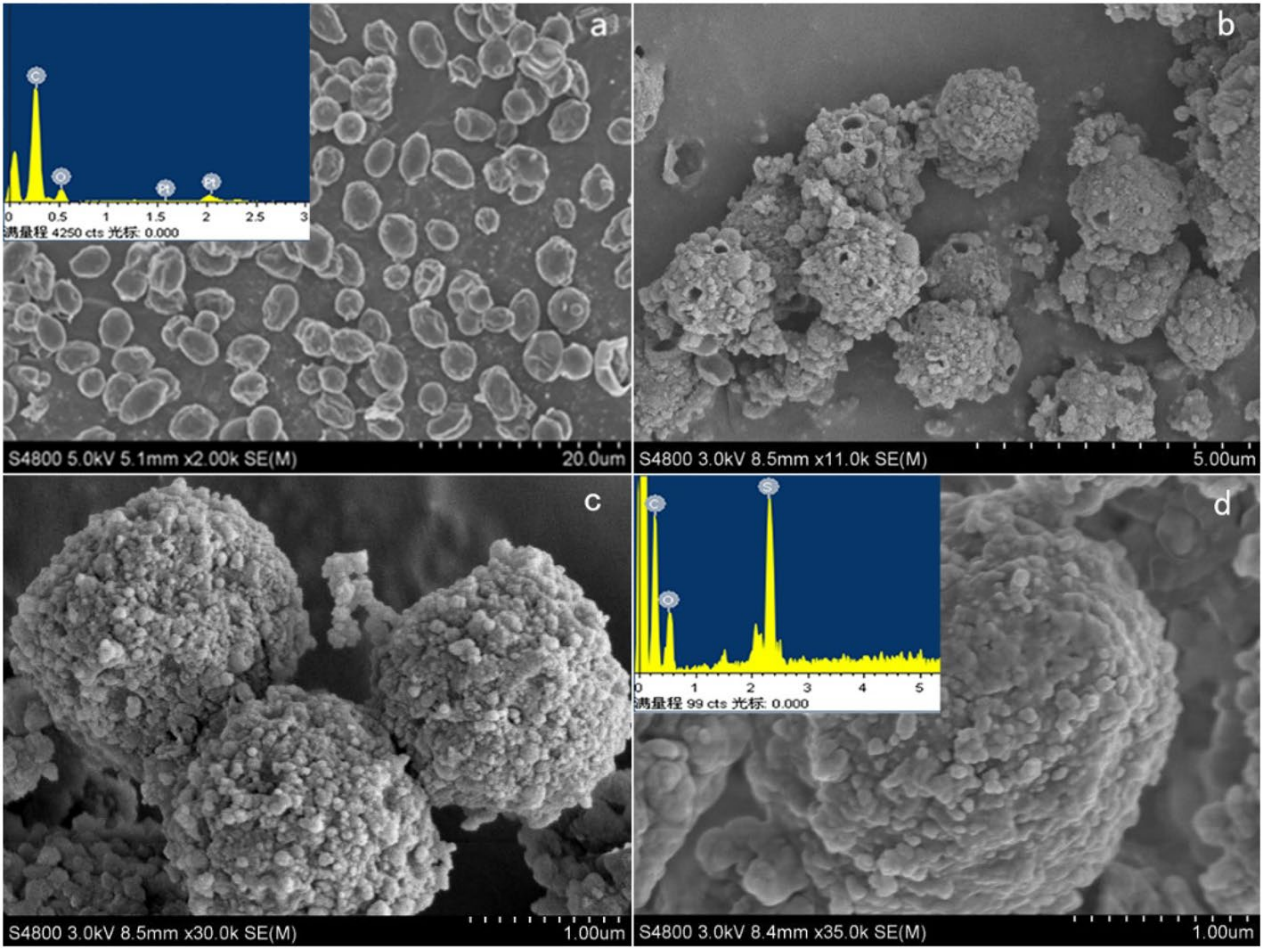
The morphology and elemental analysis of yeast and SA/YCM.

The surface features of the samples were further documented in SEM images. Figure 2 displays the surface morphology of the yeast (a), yeast carbon microspheres prepared without the addition of hydroxyethanesulfonic acid (YCM) (b), and SA/YCM (c, d), respectively.

Figure 2a presents the original structure of the yeast, and the surface was quite smooth. Figure 2b shows the morphology of the YCM formed by hydrothermal reaction at 200 ℃. The YCM have obvious sphericity and pore structure compared with the original yeast. The formation of pores occurs because the hydrolysis channels expand and combine with each other, and finally the pore structure appears in the microsphere shell. A porous structure is conducive to improving the adsorption capacity of carbon microspheres^38,45^. Further, Fig. 2c, d are the topography of SA/YCM at different magnification. Clearly, the morphology of the sulfonic acid hydrothermally modified yeast carbon microspheres is similar to that of the hydrothermally modified yeast microspheres. This result is due to the hydrothermal process after the addition of sulfonic acid does not change the morphology of the carbon microspheres, but only changes the composition of the carbon microspheres. This result can also be proven from the EDS result of the embedded graph. More specifically, hydrothermal modification did not increase the elemental composition of the carbon microspheres, but the addition of sulfonic acid groups caused the carbon microspheres to contain S.

### Interaction analyses

**Figure 3:**
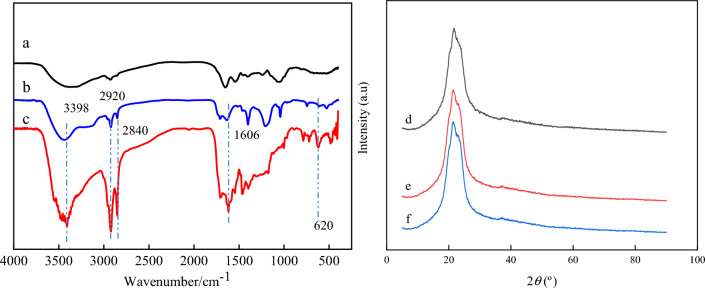
FT–IR spectrometry analysis of yeast (**a**), YCM (**b**), and SA/YCM (**c**). X-ray powder diffraction patterns of yeast (**d**), YCM (**e**), and SA/YCM (**f**).

FTIR was used to characterize the surface of the hydrothermally treated yeast to validate the chemical composition of the obtained SA/YCM. Compared to Fig. 3a, b, the spectrum of SA/YCM (Fig. 3c) displayed prominent peaks at 620 cm^–1^ attributed to sulfonic acid groups ^46^. Moreover, the prominent peaks at 3398 and 1250 cm^–1^ are attributed to –OH and C–OH stretching vibrations, respectively^47^. These evidences indicate that the product surface contains a large amount of hydroxyl. Further, the as-obtained SA/YCM displayed out of plane bending vibration peaks of C–H at 2920 and 2840 cm^−1^, demonstrating the aromatization of polysaccharide network of yeast during hydrothermal modification. In addition, the C=C absorption peak at 1606 cm^−1^ indicates that the yeast underwent carbonization during the hydrothermal process. Overall, it can be concluded that sulfonated porous yeast carbon microspheres were successfully synthesized.

To further validate that SA/YCM was synthesized, the XRD profiles of yeast, YCM, and SA/YCM were recorded, respectively. It can be seen from Fig. 3d–f, the pristine yeast was milled enough, thus resulting in a fairly low crystallinity. Namely, the bread-like diffraction peak clearly indicates that the original yeast has an amorphous structure^48^. Furthermore, the crystal structure of yeast has almost no change after hydrothermal reaction. This phenomenon shows that yeast exists in an amorphous structure before and after the hydrothermal reaction, and the amorphous structure does not change.

**Figure 4:**
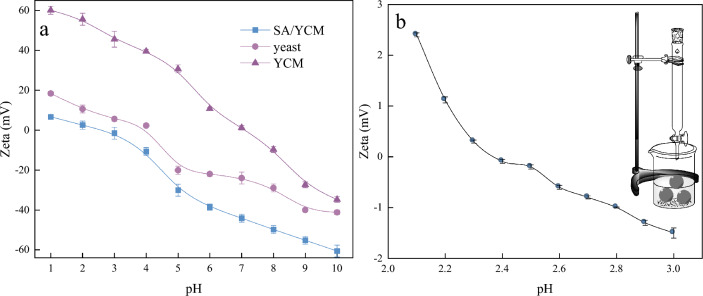
Schematic diagram for measuring the isoelectric points and zeta-potential profiles of SA/YCM at different pH values.

The Zeta potentials of the different as-obtained materials at different pH values are shown in Fig. 4a. Yeast presents a negative potential owing to the presence of hydroxyl and carboxyl groups. After modification with sulfonic acid and hydrothermal methods, SA/YCM is more electronegative when the when pH is greater than 7 compared with carbon microspheres without sulfonic group modification. Thus, the sulfonic acid group was successfully grafted onto the surface of YCM, and the electronegativity of YCM is derived from the sulfonic acid groups grafted when the pH is greater than 7 ^49,50^. More importantly, the negative potential of SA/YCM was almost less than 0 over a wide range of pH values, as shown in Fig. 4b. This fascinating characteristic originates from the charge stability of the sulfonic acid groups. To further clarify the adsorption performance of modified yeast at different pH values. From Fig. 4b, it can be concluded that the isoelectric point of SA/YCM was 2.45. Specifically, SA/YCM, which has a negative charge can adsorb cationic dyes and heavy metals through electrostatic interactions.

**Figure 5:**
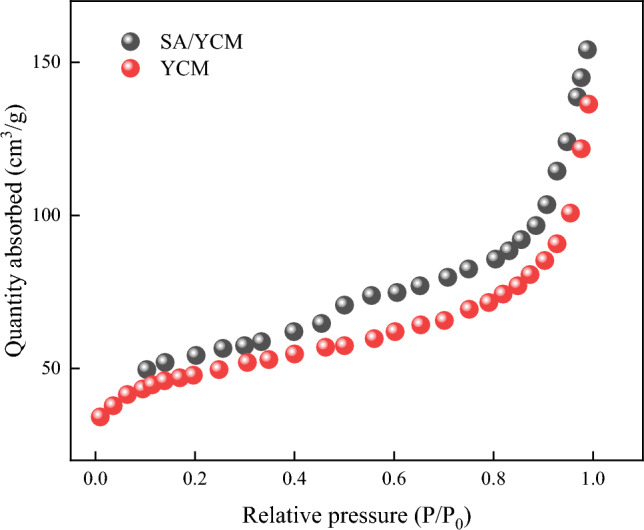
Nitrogen adsorption/desorption isotherms of YCM and SA/YCM.

The pore structures of YCM and SA/YCM were investigated by nitrogen adsorption/desorption analyses, the results of which are summarized in Fig. 5 and Table S1. The results reveal that the specific BET surface area of SA/YCM (132 m^2^/g) is higher than that of YCM (128 m^2^/g). The BJH pore volume and pore size of YCM and SA/YCM are 0.23. The results showed that the hydrothermal process endowed the carbon microspheres with a good pore structure. Meanwhile. the results show that the addition of 2-hydroxyethanesulphonic acid hardly alters the hydrothermal carbonation process.

**Figure 6:**
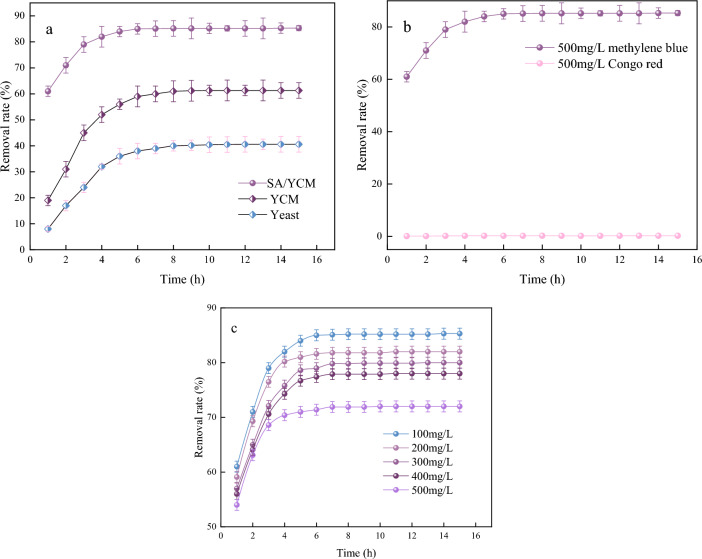
(**a**) The adsorption properties of yeast, YCM and SA/YCM. (**b**) Adsorption effect of SA/YCM on different dyes. (**c**) Effect of adsorption time on the removal rate of MB at different initial concentrations.

In consideration of practical application, we evaluate the adsorption performance of all materials. The adsorption capacities of yeast, YCM and SA/YCM are shown in Fig. 6a. It is obvious that the optimal absorbency of yeast, YCM and SA/YCM was 40.6, 61.3, 85.3 mg/L, respectively. To our best knowledge, the fabrication of carbon microspheres can improve the pores and storage sites of materials. Sticking to this principle, the hydrothermal reaction of yeast destroys yeast cells and carbonizes them, which makes the yeast rough and improves its pores and specific surface area^51^. Hydrothermal modification can greatly improve the adsorption performance of yeast. Additionally, adding 2-hydroxyethanesulphonic acid during hydrothermal process can synthesize negatively charged, which improve adsorption performance of cationic dyes. Considering this excellent property, the adsorption performance of SA/YCM is higher than that of unmodified yeast and YCM.

**Figure 7:**
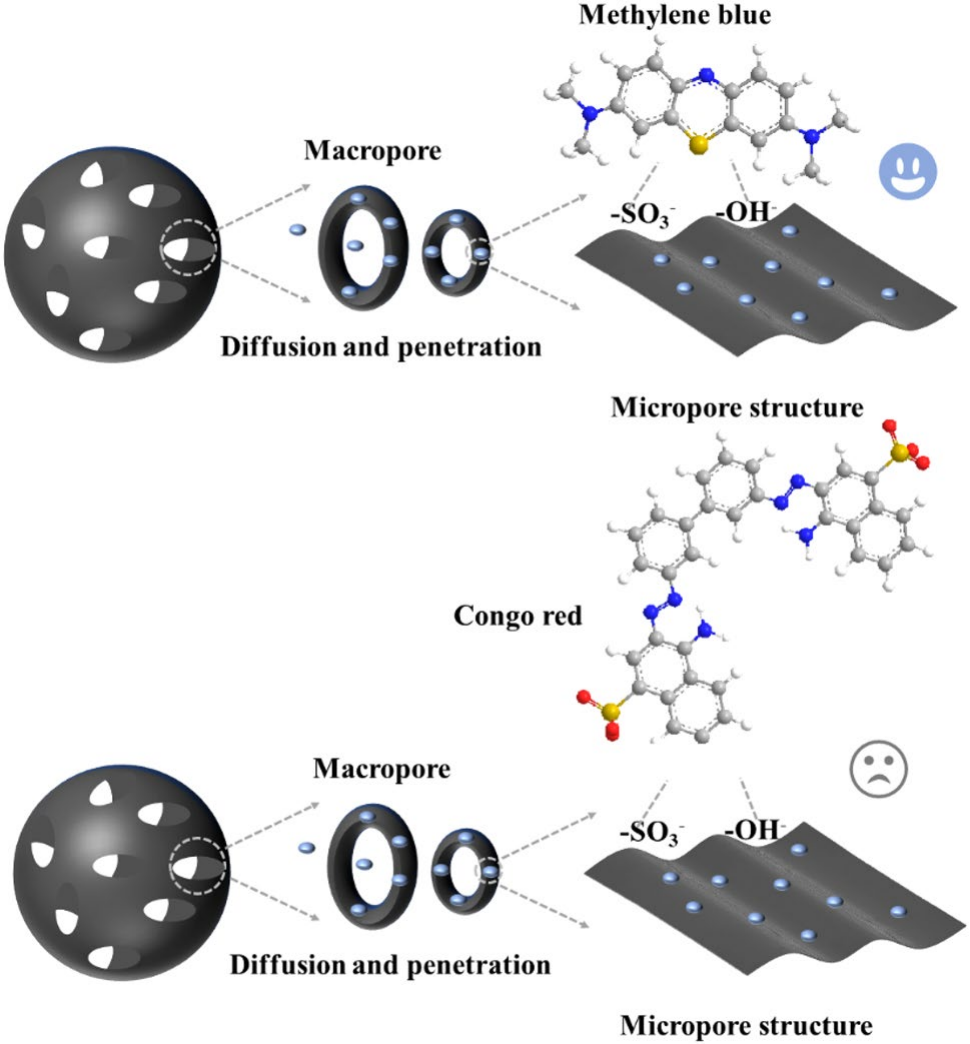
The selective adsorption mechanism of SA/YCM.

The ability of SA/YCM to remove different dyes was evaluated in a 500 mg/L aqueous solution (Fig. 6). Adding SA/YCM to Congo red solution led to almost 0.3% Congo red removal. This phenomenon occurs because the SA/YCM surface contains abundant -OH and -SO_3_H, which strongly repel negatively charged Congo red (Fig. 7). Thus, Congo red cannot be adsorbed to the interior of the microspheres, even though the microspheres have a large number of pores. To further clarify the adsorption performance of the cationic dyes, the MB adsorption performance of SA/YCM was recorded in Fig. 6b. From the adsorption results, the adsorption capacity gradually increased and reached equilibrium after 6 h. Such good adsorption phenomenon could be attributed to the binding of cationic dyes to –OH and –SO_3_H on SA/YCM, resulting in chemical adsorption. What’s more, the pore structure of SA/YCM can also rapidly adsorb MB through concentration gradient. The adsorption results indicated that synergy results from the adsorptive properties of the porosity and large amount of negative charges on the surface, which increases the adsorption effect. Based on the above phenomenon, we can draw the conclusion that the sulfonic acid group modification and the porosity of the carbon microspheres clearly demonstrated the efficient cationic dye removal capacity of the obtained SA/YCM. To further test the ability of SA/YCM to adsorb cationic dyes, treatment of MB wastewater with different concentrations by using SA/YCM and the results were recorded in Fig. 6b. As can be seen from Fig. 6c, the cumulative absorption rate of MB increased with time and finally reached equilibrium. Furthermore, the adsorption rate decreased with increasing of MB concentration. This phenomenon is due to the adsorption capacity of SA/YCM reached saturation.

**Figure 8:**
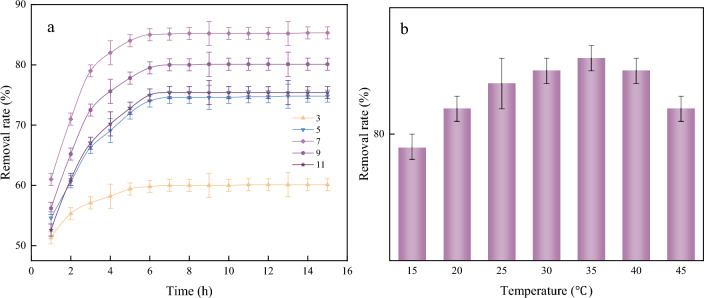
Effect of different pH values (**a**) and temperatures (**b**) on the removal rate.

As shown in Fig. 8a, the highest removal rate of SA/YCM for MB was 85.3%. The adsorption capacity of SA/YCM was weak under acidic conditions, which was mainly due to the protonation of SA/YCM and thus difficulty in adsorbing MB^52^. The surface dipole layer changed the polarity of SA/YCM when the pH of the solution became neutral, resulting in an increase in the removal rate of MB. However, when the solution became strongly alkaline, increasing the ionic strength of the system led to a decrease in the adsorption effect.

To clarify the adsorption behavior at different temperatures, adsorption tests of SA/YCM were carried out at different temperatures. As presented in Fig. 8b, the dye removal rate increased gradually with increasing of temperature from 15 to 35 °C, which was attributed to the increased adsorptive molecular activity and adsorptive site activity, as well as the increased rate of controlled diffusion of adsorbate molecules in the adsorbent. However, the removal rate of SA/YCM decreased when the temperature exceeded 35 °C, which may be due to excessive intermolecular strength between system molecules resulting in a decrease in the removal rate. Overall, the adsorption research results showed that removal rate was as high as 86% at 35 °C.

**Figure 9:**
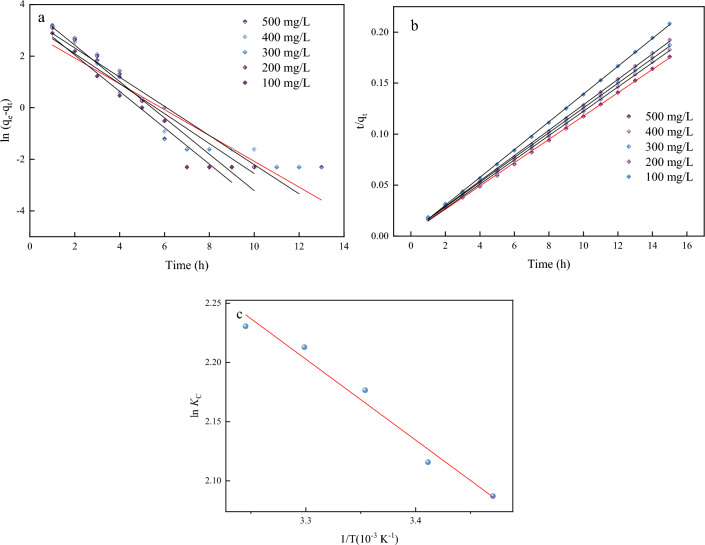
Absorption kinetics of SA/YCM on MB at different concentrations (**a,b**). The thermodynamics model of SA/YCM on MB at different temperature (**c**).

The parameters obtained from the kinetics models are presented in Table 1 and Fig. 9. It is evident from Table 1 that a more precise fit of kinetics data was shown by the pseudo-second-order model for dye adsorption. The values of MB calculated adsorption capacity are much closer to the experimental adsorption capacity for pseudo-second-order kinetic model and the values of regression coefficients (*R*^2^) are higher than pseudo-first-order kinetic model. This result is due to the pseudo-first-order model is suitable for elucidating the physical adsorption (yeast carbon microspheres adsorption methylene blue). However, the MB adsorption through SA/YCM was governed by multiple factors (physical adsorption and chemical adsorption process). Namely, sulfonic groups and hydroxyl groups on the surface of the SA/YCM can chemically adsorb MB, which endows the carbon microspheres with chemical adsorption capacity. Hence, the pseudo-second-order model is suitable for describing the cationic dye absorption process of SA/YCM.

**Table 1:** Adsorption kinetic parameters of SA/YCM on MB at different concentrations.

*C*_0_ (mg/L)	*Q*_e_ (mg/L)	Pseudo-first-order model			Pseudo-second-order model		
		*q*_e_ (mg/g)	*k*_1_ (min^−1^)	*R*^2^	*q*_e_ (mg/g)	*k*_2_ (min^−1^)	*R*^2^
100	72.0	35.4	0.50	0.83498	73.4	0.039	0.9996
200	156.0	106.3	0.58	0.89487	160.6	0.045	0.9996
300	240.0	229.8	0.57	0.91771	247.5	0.036	0.9996
400	328.0	300.1	0.70	0.92809	335.9	0.039	0.9996
500	426.5	402.2	0.70	0.95607	437.8	0.062	0.9998

The thermodynamic parameters for the adsorption of MB on SA/YCM at 288.15, 293.15, 298.15, 303.15, and 308.15 K are exhibited in Table 2. Negative Δ*G* and positive Δ*S* values elucidate that the adsorption takes place spontaneously. The absolute values of Δ*G* are lower than 20, which clarifies that the physical adsorption is the dominant factor in the whole adsorption process. Additionally, the positive Δ*H* indicates the removal process is endothermic, representing that the physical interaction as well as pore diffusion and mass transfer predominate in the whole removal process^53^.

**Table 2:** Thermodynamic parameters for removal MB onto SA/YCM.

T	Δ*G* (KJ/mol)	Δ*H* (KJ/mol)	Δ*S* [J/(mol·K)]
288.15	−4.99	5.69	37.08
293.15	−5.16		
298.15	−5.40		
303.15	−5.58		
308.15	−5.71		

Reusability is a significant feature of adsorptive materials in controlling dye pollution in water^54^, notably mechanical friction may destroy the surface micro-structures, and even decreases the content of sulfonic acid group. To verify the durability of SA/YCM, here, the simple and common adsorption-regeneration experiment was conducted to test the durability of SA/YCM through the utilize of acid to regenerate the SA/YCM ^55^.

**Figure 10:**
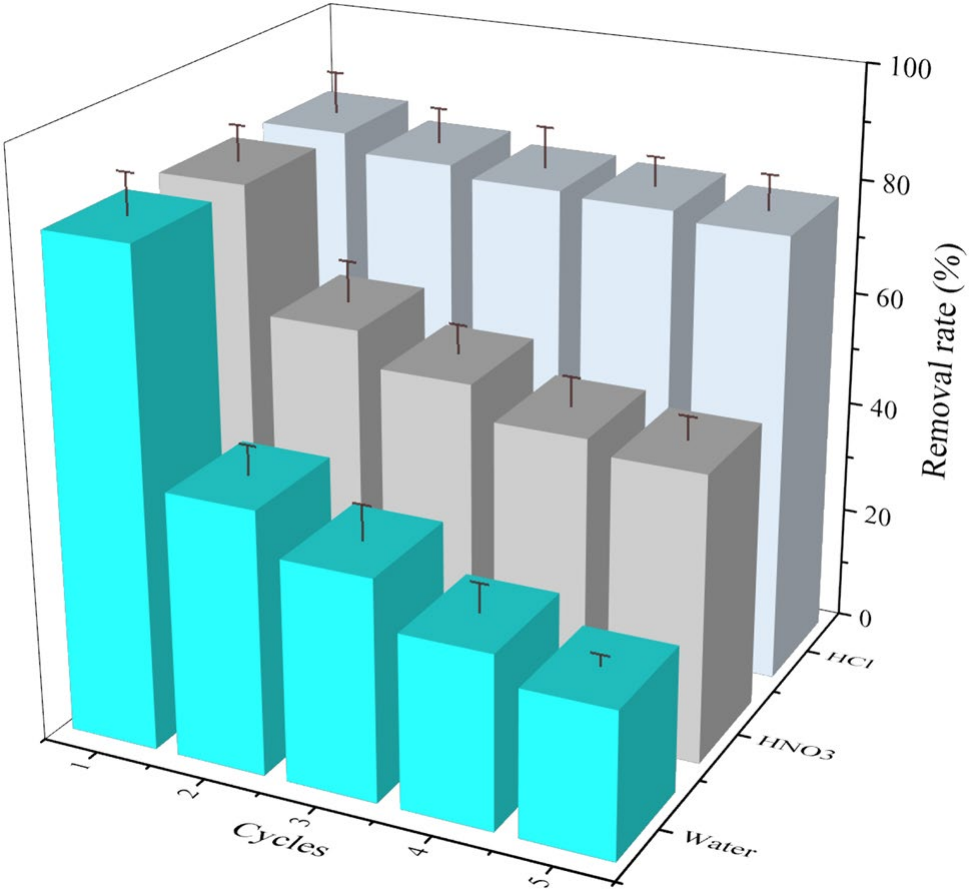
The experimental results of regeneration of MB adsorbed by SA/YCM.

Hereby, as illuminated in Fig. 10, the regeneration of obtained SA/YCM was conducted by adding deionized water, HCl and HNO_3_, which is defined as one cycle. The SA/YCM regenerated by HCl showed a more stable regeneration adsorption capacity after five cycles of adsorption–desorption regeneration. The adsorption rate of MB on SA/YCM regenerated by HNO_3_ decreased to 49.8%, which may be due to HNO_3_ etching the internal pore structure of SA/YCM, destroying the structure of micropores, and decreasing the adsorption efficiency.

Table 3 summarizes the comparison of preparation methods and adsorption capacity of various adsorbents for MB. Compared with previous materials, SA/YCM showed relatively high adsorption capacity for MB. Therefore, it can be inferred that the surface charged properties have undergone significant changes, which should be attributed to the unique mechanism of sulfonic acid modification. In SA/YCM, hydrothermal carbonization provides yeast with a larger pore structure and Adsorption site. Moreover, the hydrothermal method is simple to operate and cost-effective, providing a new approach for the treatment of dye wastewater.

**Table 3:** Comparison of the adsorption capacity and preparation methods of different adsorbents for MB.

Samples	Preparation method	Comparison	Adsorption capacities for MB	References
SA/YCM	Hydrothermal	Cheap raw materials and hydrothermal treatment only	426.5 mg/g in 500 mg/L wastewater	This work
PVA CACms	Hydrothermal and chemical activation	Added subsequent material activation	602.4 mg/g in 1000 mg/L MB wastewater	^56^
Pomelo skin activated carbon	Microwave-assisted preparation and chemical activation	Complex preparation methods	332.87 mg/g in 500 mg/L MB wastewater	^57^
Rattle-type magnetic carbon nanospheres	Hydrothermal and annealing treatment	Added annealing process	45.15 mg/g in 1000 mg/L MB wastewater	^58^
Raspberry-like microspheres	electrostatic-interaction-driven selfassembly method	The self-assembly process is difficult to control	397.97 mg/g in 125 mg/L MB wastewater	^59^

## Conclusion

In conclusion, we have demonstrated a sulfonate modified yeast microspheres by a hydrothermal method. Utilizing the hydrothermal method, the yeast tends to dehydrate within the molecule, and a large amount of gas in the cell penetrates the microsphere shell to form porous carbon morphology. Meanwhile, the obtained combination of sulfonic acid group, were firmly attached to the surface of yeast carbon by hydrothermal process. The obtained SA/YCM can be used to adsorb cationic dyes in wastewater. More importantly, owing to the mechanical properties of carbon microsphere and stable adhesion of sulfonic acid groups, the SA/YCM showed prominent mechanical durability, which could withstand repeated adsorption- desorption cycle as well as multiple practical applications. Also, the above prominent adsorption performance and durability are prospective candidate for the replacement of traditional neutral adsorption materials.

### Supplementary Information

Supplementary Table S1.

## Data Availability

The datasets generated and/or analysed during the current study are available in the science data bank repository, https://www.scidb.cn/anonymous/YlV6aVV2.
