# Supplementary material for: Facile fabrication of sulfonated porous yeast carbon microspheres through a hydrothermal method and their application for the removal of cationic dye
Source: Sci Rep. 2024 May 17;14:11326. doi: 10.1038/s41598-024-62283-w (PMC11101640; doi:10.1038/s41598-024-62283-w)
Supplement: Supplementary file 1 — Supplementary Table S1. [file 41598_2024_62283_MOESM1_ESM.docx]

**Facile fabrication of sulfonated porous yeast carbon microspheres through hydrothermal method and their applications for removal of cationic dye**

Yang Chenxi * ^a, b, c, d^, Zhang Haiou ^a, b, c, d^, Wang Jian ^a, b, c, d^, Wang Yingguo ^a, b, c, d^

a. Institute of Land Engineering and Technology, Shaanxi Provincial Land Engineering Construction Group Co., Ltd., Xi'an 710075, China.

b. ShaanXi Provincial Land Engineering Construction Group Co., Ltd., Xi'an 710075, China.

c. Key Laboratory of Degraded and Unused Land Consolidation Engineering, the Ministry of Natural Resources. Ltd., Xi'an 710075, China.

d. Shaanxi Provincial Land Consolidation Engineering Technology Research Center. Xi'an 710075, China.

* Corresponding author

Email address: 1098002212@qq.com

**Table S1**. BET Surface Area and BJH Pore Volume, Pore Size Summary for Different Samples

|  | S_BET_  (m^2^/g) | BJH pore volume  (cm^3^/g) | Average BJH pore (cm^3^/g) |
| --- | --- | --- | --- |
| YCM | 128 | 0.23 | 4.9 |
| SA/YCM | 132 | 0.23 | 4.9 |
